# Evaluation of Clareon Vivity and PureSee intraocular lenses: optical quality, depth of focus and misalignment effects

**DOI:** 10.1038/s41598-025-07970-y

**Published:** 2025-07-24

**Authors:** Ava Niknahad, Zhiyi Wu, Hyeck-Soo Son, Gerd U Auffarth, Ramin Khoramnia, Grzegorz Łabuz

**Affiliations:** 1 Department of Ophthalmology, The David J. Apple Center for Vision Research, University of Heidelberg, 69120 Heidelberg, Germany; 2 Eye Center, The Second Affiliated Hospital, Zhejiang University, Hangzhou, 310009 China; 3 Department of Ophthalmology, University Hospital Carl Gustav Carus, TU Dresden, 01307 Dresden, Germany

**Keywords:** Extended depth of focus, Intraocular lens, Clareon vivity, Tecnis puresee, IOL misalignment, Applied optics, Eye diseases, Lens diseases, Materials for optics

## Abstract

Extended depth of focus (EDoF) intraocular lenses allow cataract and refractive-lens-exchange patients to have improved vision at an intermediate distance. Two EDoF lenses, Clareon Vivity and Tecnis PureSee, were evaluated for optical quality using the OptiSpheric IOL Pro2 according to ISO 11979-2 guidelines and measured using Modulation Transfer Function (MTF) and 1951 USAF resolution test chart at 3.0- and 4.5-mm apertures. The IOLs’ tolerance to 0.50 mm decentration and a 5° tilt were tested. The unwanted visual effects were compared using their light distribution at 4.5 mm aperture beyond a polychromatic point spread function center. The two models demonstrated comparable optical qualities at intermediate and far focuses; the predicted visual acuities for the Vivity and PureSee were respectively − 0.04 versus − 0.05logMAR at 0 diopter and 0.05 versus 0.07logMAR at − 1.50 diopters of defocus. Increasing pupil size decreased both lenses’ optical quality. While decentration decreased the optical quality of both models, tilt had less of an effect. Both were similar in introducing minimal unwanted side effects through visualizing a halo pattern. The results indicate that these lenses offer a good option for patients interested in EDoF lenses and future clinical studies should contribute further insight into their performance and side effects.

**Supplementary Information:**

The online version contains supplementary material available at 10.1038/s41598-025-07970-y.

## Introduction

With the development and use of internet-connected devices for work and entertainment, many individuals spend a significant portion of their days looking at screens. For example, the average screen time for users in the United States of America has been estimated to be 7.1 h, in the United Kingdom to be 7.2 h, and globally 6.7 h per day^1,2^. Individuals often have an average viewing distance of 54 cm to 62 cm when looking at a computer^3,4^, and 35 cm in non-presbyopes, and 39 cm in presbyopes when looking at smartphones^5^. Apart from these examples of screen viewing, shopping, conversing, eating, controlling the car dashboard, or organizing desks are all activities that rely on proper visual acuity (VA) across distances of 50 cm to 100 cm, termed intermediate vision^6^.

Given such reliance on intermediate vision in daily life, developing intraocular lenses (IOLs) that allow spectacle-free vision at this distance has been increasingly crucial for individuals undergoing crystalline lens replacement. Extended depth of focus (EDoF) lenses allow this improved visual quality, especially at the intermediate distance, by splitting the light into a continuous and elongated area of focus^7–9^. By having continuous rather than discrete areas of focus and by using the additional principles of negative spherical aberration or the pinhole effect, and diffractive or refractive designs, different EDoF IOL designs have been developed with properties differently affecting various aspects of visual function^7,9–11^.  A prospective study by Pedrotti et al. comparing clinical outcomes of an aspheric monofocal IOL to an EDoF IOL found significantly better uncorrected monocular and binocular distance, intermediate, and near visual acuities for patients implanted with the EDoF IOL compared to monofocal IOLs^8^. Recent reviews have demonstrated that halos and glare are more common in diffractive than in refractive EDoF IOLs^12^. In a comparative study of trifocal IOLs with EDoF IOLs, Zhong et al. reported better near and far VA with trifocals but better intermediate vision with the EDoF. They additionally reported the halo side-effect to be more common with trifocals, while contrast sensitivity and subjective visual quality were comparable between the two groups^13,14^. Lastly, a meta-analysis published in 2024 by Tavassoli et al. on studies comparing trifocal and EDoF IOLs in patients undergoing cataract surgery revealed that overall, those receiving trifocal IOLs may have better near vision, but distance VA, glare, and halos are similar between the two lenses.

While many studies, such as those mentioned, have evaluated EDoF lenses, laboratory evaluations of optical quality are still needed for new models, such as the Tecnis ZEN00V/DEN00V PureSee (Johnson & Johnson Vision Inc., Irvine, CA, USA) and the Clareon Vivity (Alcon Laboratories, Fort Worth, TX, USA). In addition to their newer designs, these two IOLs are popular EDoF choices, with Clareon Vivity being reported as the most implanted EDoF IOL globally in 2023 and Johnson & Johnson being the most popular brand for its one-piece Tecnis monofocal in a survey of 51 surgeons in 2024^15,16^. Laboratory studies on these lenses can therefore form an important baseline for understanding future clinical studies of these lenses. Moreover, the IOLs’ tolerance to misalignment and changing spectral and spherical-aberration (SA) conditions were also investigated.

## Results

### Modulation transfer function (MTF) analysis

**Fig. 1:**
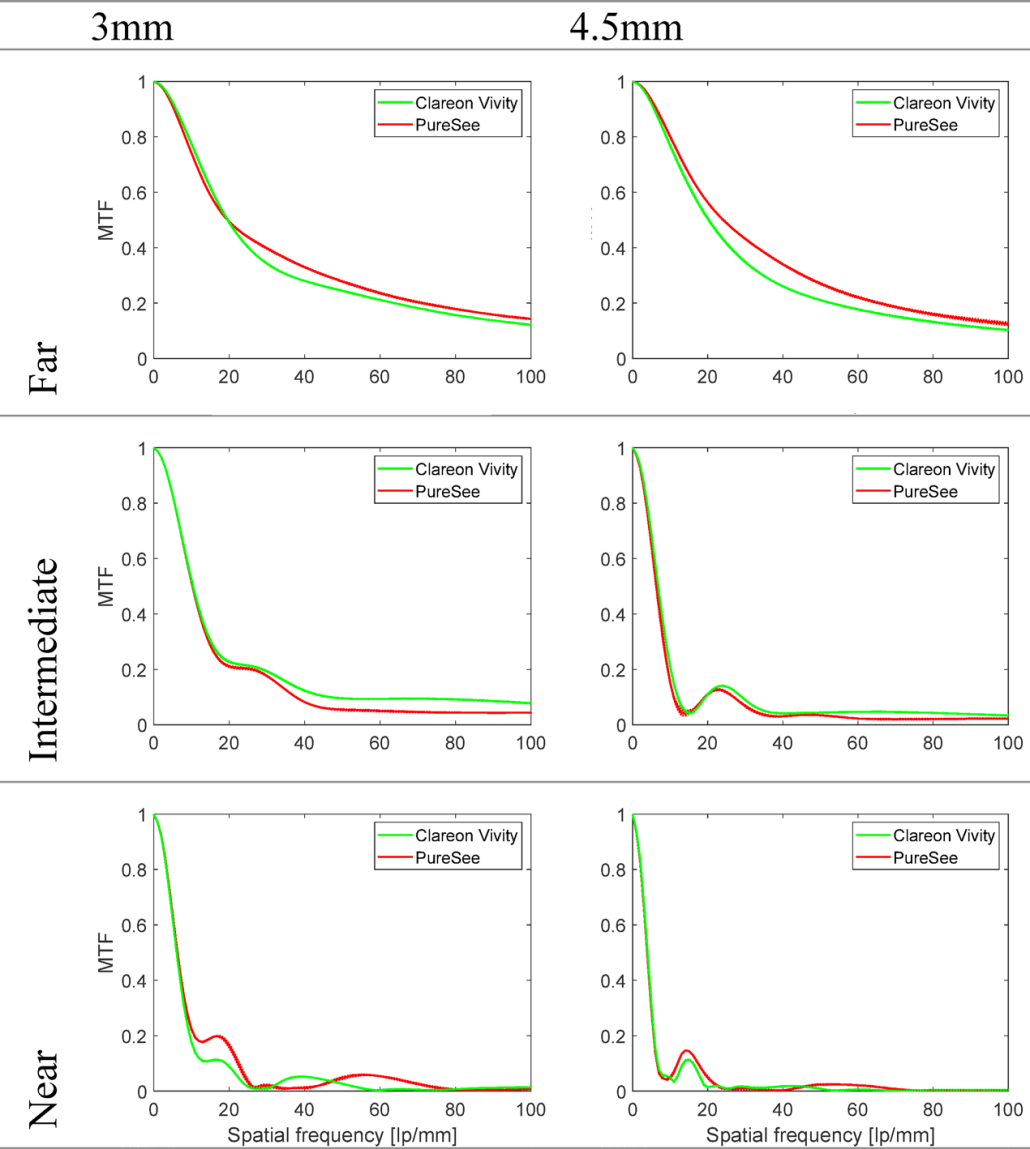
Modulation transfer function (MTF) levels of the intraocular lenses at the best-far focus MTF, along with MTFs obtained at − 1.5D and − 2.5D corresponding to intermediate and near distances at 3- and 4.5-mm aperture sizes. The dotted lines show the values of each lens separately; the solid lines refer to the average of two samples. Both models showed comparable performance at far focus, with PureSee performing slightly better. At intermediate foci, Vivity showed superior performance to PureSee. Both lenses’ performance decreased with increasing pupil size.

Figure 1 shows the average MTF curves of the studied IOLs at 3- and 4.5-mm. At 3 mm and the far point, both models showed comparable performance, with the Vivity having a slightly lower mean (± standard deviation) MTF value at 50 lp/mm (0.24 ± 0.00) compared to the PureSee (0.28 ± 0.00). At intermediate distances, the Vivity showed slight improvement over the PureSee, with an MTF of 0.10 ± 0.00 at 50 lp/mm, while the PureSee had an MTF of 0.05 ± 0.00. Both IOLs exhibited low MTF values at near distances, with the Vivity at 0.03 ± 0.00 and the PureSee at 0.05 ± 0.00 at 50 lp/mm. At 4.5 mm, the MTF curves of the Vivity were slightly below those of the PureSee with an MTF at 50 lp/mm of 0.21 ± 0.00 compared to 0.27 ± 0.00. Both lenses showed reduced optical quality at intermediate and near distances, with MTF values at 50 lp/mm ranging from 0.03 to 0.04 at intermediate and from 0.01 to 0.02 at near.

### Through-focus (TF) MTFs

**Fig. 2:**
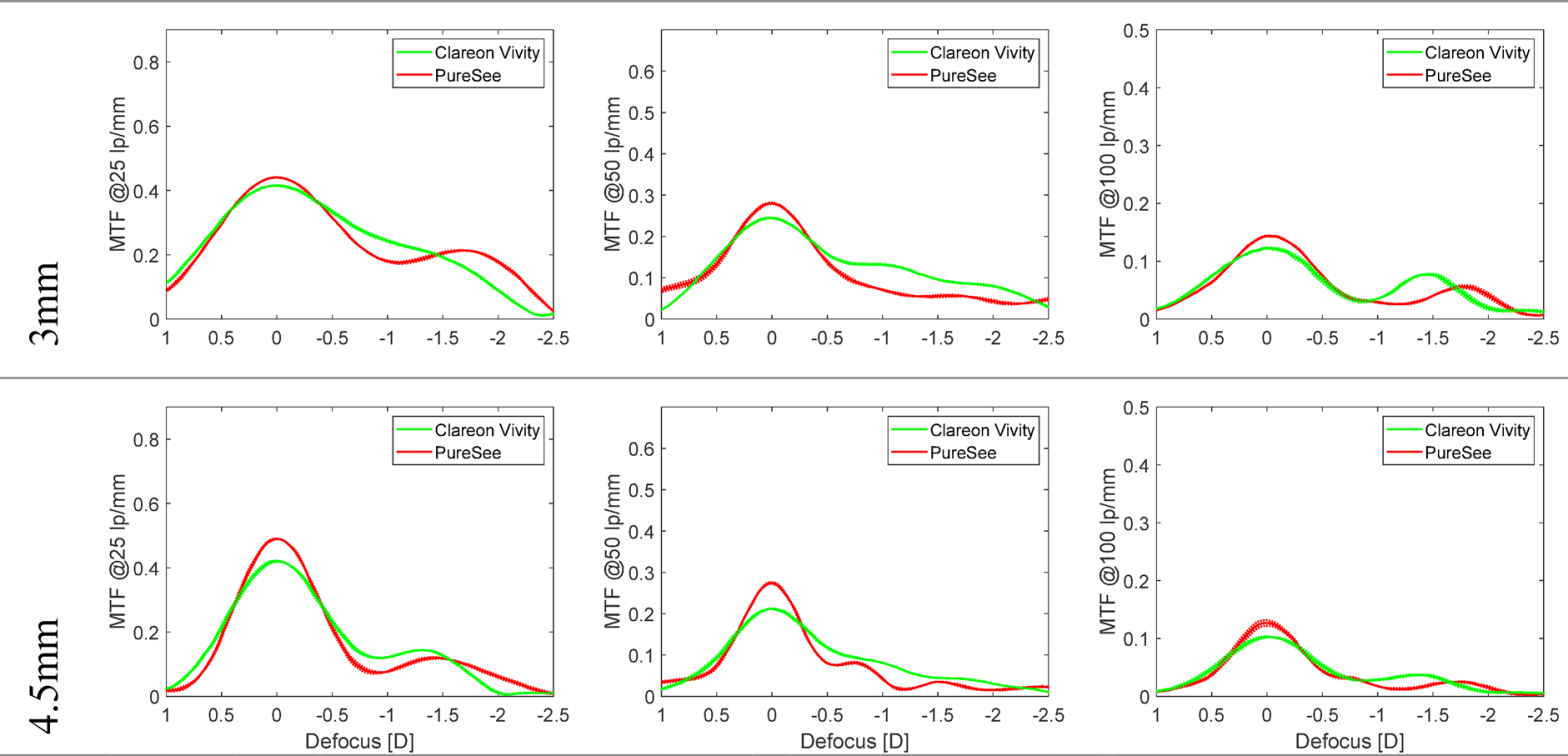
The through-focus modulation transfer function curves of the studied models were measured at three discrete spatial frequencies of 25, 50, and 100 lp/mm. The dotted lines show the values of each lens separately; the solid lines refer to the average of two samples. Overall Vivity demonstrated an EDoF effect beginning 25 lp/mm while PureSee contained a secondary peak at −1.75D. PureSee gained a plateau in its peak position at 50 lp/mm. By 100 lp/mm, both models had a secondary peak again.

Figure 2 reports the TF MTF of the tested models measured at 25, 50, and 100 lp/mm and the two apertures.

At 3 mm and 25 lp/mm, the Vivity demonstrated an EDoF effect, while the PureSee produced a secondary extended peak at about − 1.75 D, which can also be seen at 100 lp/mm. At 50 lp/mm, the Vivity and PureSee yielded a plateau, with slightly higher values of the Vivity at an extended defocus range. At 100 lp/mm, both lenses presented with the MTF peaks, which were shifted toward higher negative defocus for the PureSee. The presence of two distinguishable peaks may indicate the design characteristics of both IOLs, reflecting their effective add power generated by their complex designs and highlighting the optically provided range of vision. The aperture size increase affected the IOLs’ tolerance to defocus, showing overall lower values compared to the 3 mm assessment.

### United States Air Force (USAF) resolution test, area under the MTF curve (MTFa), and visual acuity (VA) simulations

**Fig. 3:**
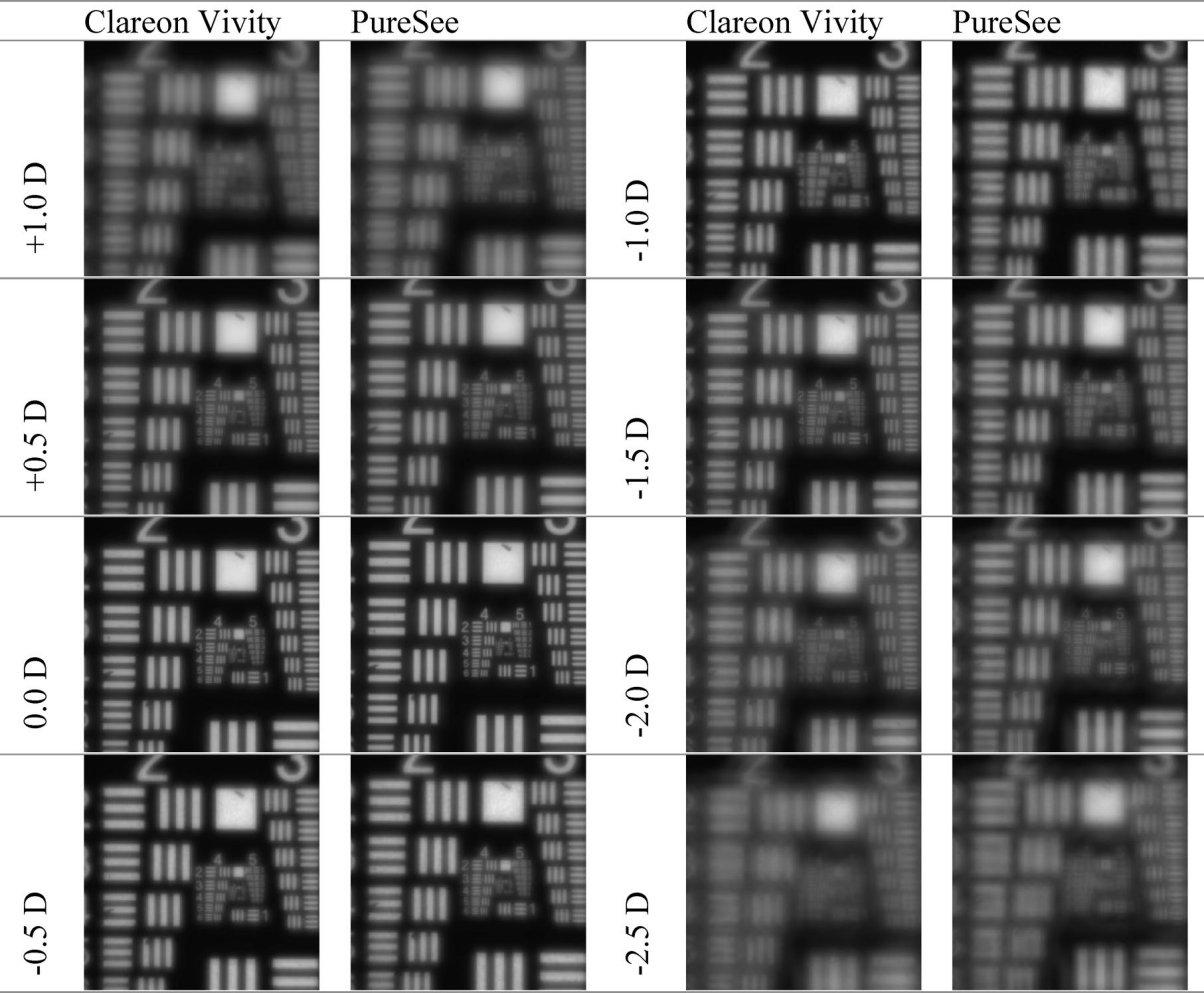
United States Air Force resolution targets recorded at a defocus range of + 1.0 to − 2.5D and the 3-mm aperture. Both models showed images with good resolution at the intermediate distance.

**Fig. 4:**
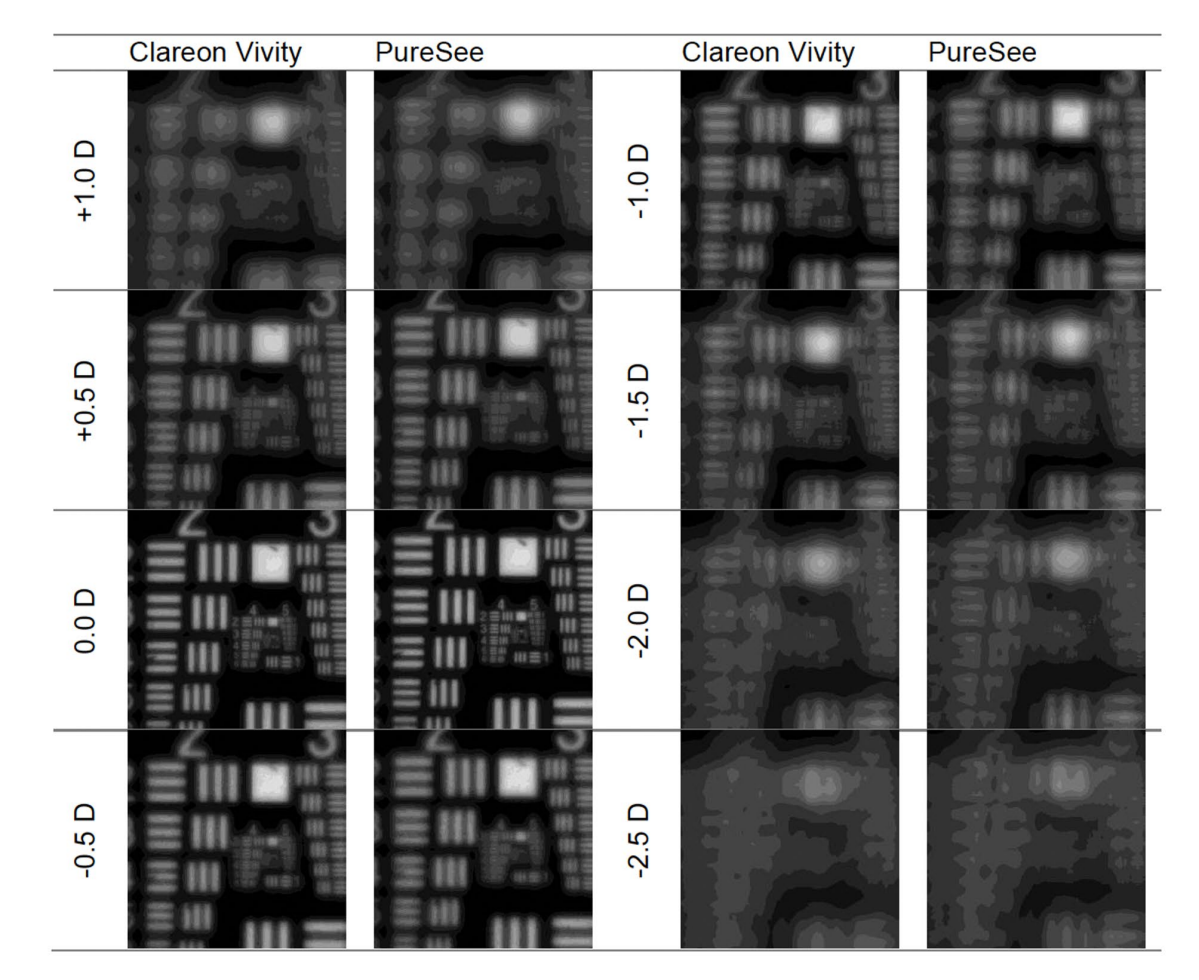
United States Air Force resolution targets recorded at a defocus range of + 1.0 to − 2.5D and the 4.5-mm aperture. While both models showed good image contrast at the intermediate focus, the image resolution overall decreased with the 4.5-mm aperture compared to the 3-mm aperture.

The resolution-test images are presented in Fig. 3 for the 3 mm aperture, and in Fig. 4 for the 4.5 mm aperture.

**Fig. 5:**
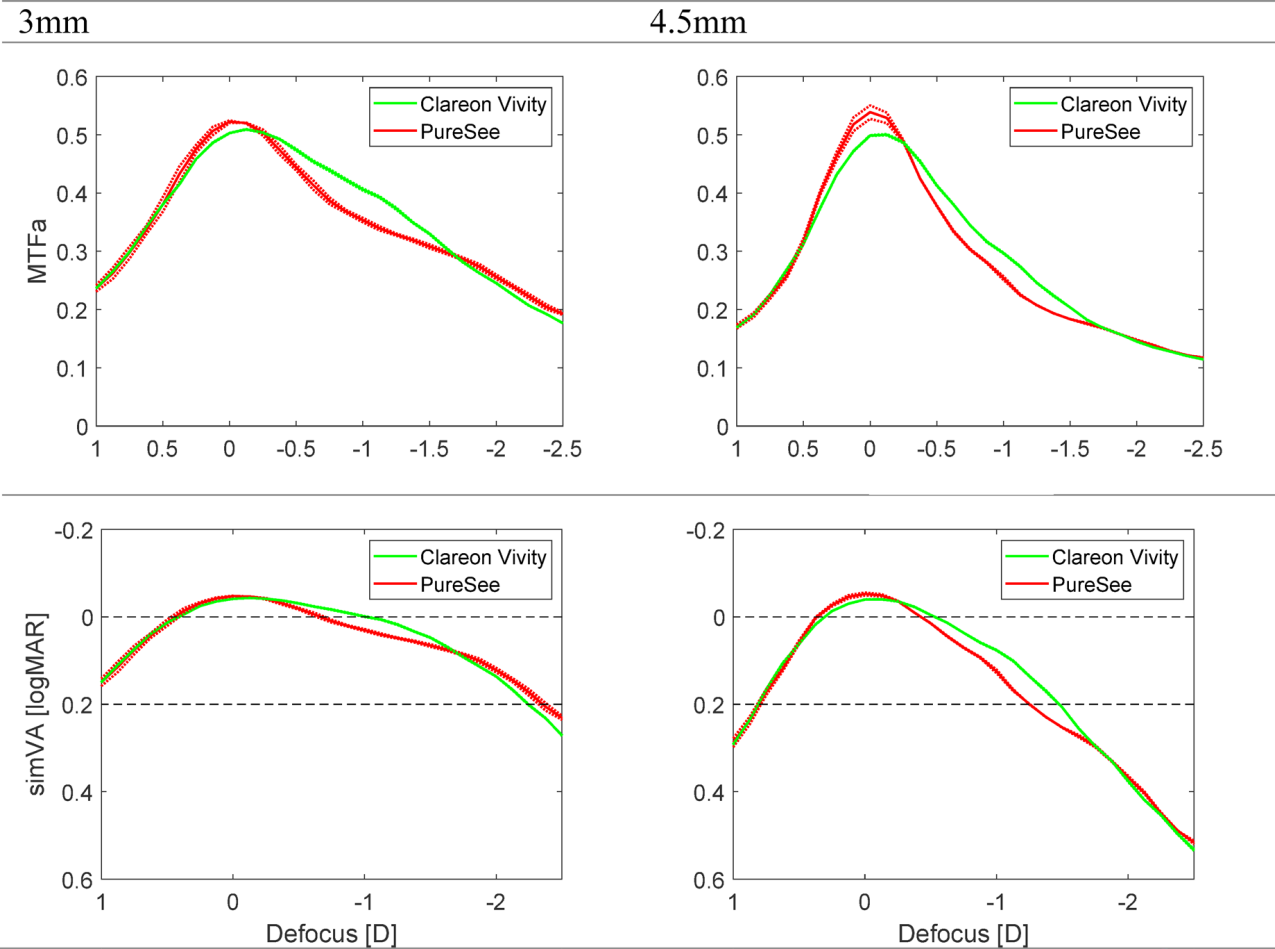
The average modulation transfer function (MTFa) value of the studied intraocular lenses is presented in the upper panel and simulated visual acuity (simVA) is presented in the lower panel as measured at the defocus range from + 1D to − 2.5D at the spectacle plane. The dotted lines show the values of each lens separately; the solid lines refer to the average of two samples. Both models demonstrate their EDoF characteristics, with PureSee containing a higher primary peak and Clareon Vivity having a higher performance in the intermediate focus value of − 1D in both pupil sizes. Pupil size increase resulted in a narrow depth-of-focus extension of the primary peak for both models.

The MTFa change for the three models at 3 and 4.5 mm can be found in Fig. 5. Both IOL models demonstrate their EDoF characteristics, with the PureSee showing a higher primary peak. However, the Vivity performed better at intermediate distance, corresponding to about − 1D of defocus, irrespective of the aperture. Defocus curves derived from the MTFa are presented in the lower panel of Fig. 5. At 3 mm, the studied IOLs had a simulated VA (simVA) of 0.2 logMAR or better throughout a range of + 1D to −2.25D, with the PureSee having this point extended by − 0.10D. At 0D of defocus, the predicted VA was − 0.04 logMAR for the Vivity and − 0.05 logMAR for the PureSee. At −1.50D, the Vivity demonstrated a simVA of 0.05 logMAR, compared to 0.07 logMAR of the PureSee, while at near, it was 0.28 logMAR and 0.24 logMAR, respectively. These differences in VA translate to less than a one-letter difference on the Snellen chart. The aperture size increase resulted in a narrower depth-of-focus extension of the primary peak for both models, with the two IOLs falling below the 0.2 logMAR mark at approximately − 1.25D (PureSee) and − 1.50 (Vivity).

## IOL decentration

**Fig. 6:**
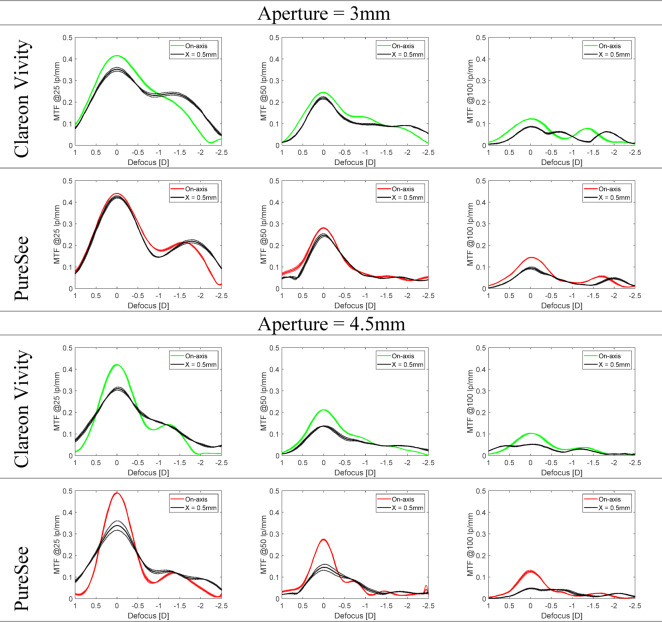
The through-focus modulation transfer function at 25, 50, and 100 lp/mm measured at on- (colored lines) and off-axis (black lines). The dotted lines show the values of each lens separately; the solid lines refer to the average of two samples. Decentration lowered the optical performance as determined by the primary peak for both models, and impacted the EDoF properties, especially for Clareon Vivity at 3-mm aperture at 25 lp/mm.

Figure 6 presents a discrete MTF value at 25, 50, and 100 lp/mm as a function of defocus measured before and after IOL decentration by 0.50 mm for 3- and 4.5-mm apertures.

At both apertures, the effects of decentration were noticeable, particularly at 4.5 mm, where it substantially lowered the primary peak for both models. At 3 mm, although decentration had a lesser impact on optical quality, it still caused a noticeable shift in the secondary (intermediate) point of the Vivity and the PureSee, which was evident at 25 and 100 lp/mm.

## IOL Tilt

**Fig. 7:**
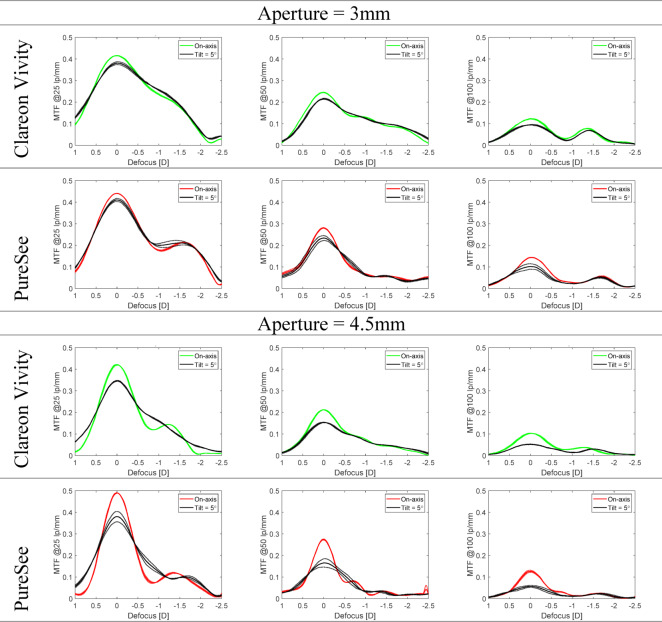
The through-focus modulation transfer function at 25, 50, and 100 lp/mm measured at no tilt (colored lines) and 5° tilt (black lines). The dotted lines show the values of each lens separately; the solid lines refer to the average of two samples. Tilt resulted in a slight reduction of both model’s primary peak while preserving their EDoF properties, especially at 3-mm aperture.

The tilt effects on the TF MTF at 25, 50, and 100 lp/mm are presented in Fig. 7. By contrast to decentration, tilt resulted in a slight reduction of the primary peak of the two IOLs without affecting the EDoF performance at 3 mm. More pronounced changes were observed at 4.5 mm, which mainly affected far-focus performance.

## Testing of unwanted visual effects

**Fig. 8:**
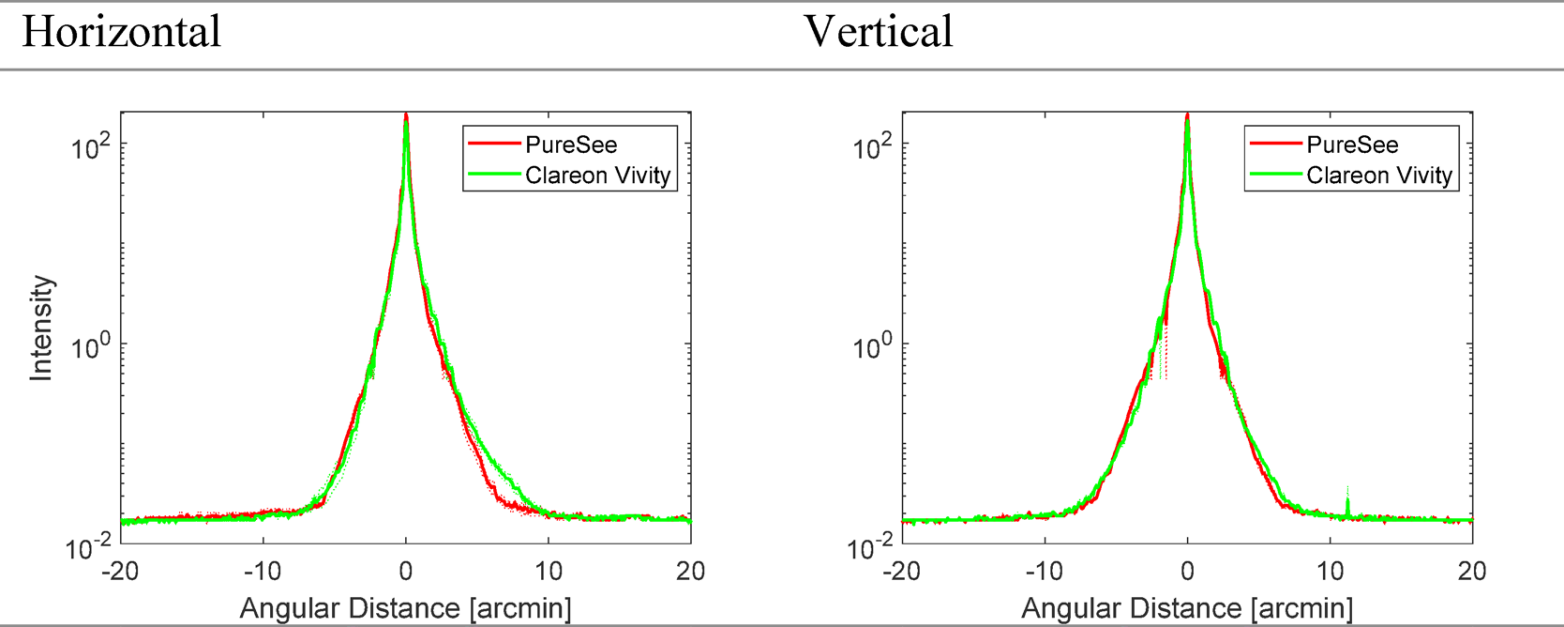
The horizontal (left panel) and vertical (right panel) cross-section of the PSF’s intensity profile for the intraocular lens models. The dotted lines show each lens’ values separately; the solid lines refer to the average of two samples. The two models demonstrated a nearly identical side effect of halo-like pattern production.

Figure 8 presents the Point Spread Function (PSF) cross-sections of the two models. The PureSee demonstrated a minimally higher peak intensity than the Vivity. Both lens models demonstrated a nearly identical PSF spread without discontinuities that are characteristic of a halo-like pattern.

## Discussion

We found that the Clareon Vivity and the PureSee EDoF IOLs have comparable optical qualities at far focus, with predicted VA exceeding 20/20 (0.00 logMAR). Despite their differences in the optical designs, both models have the potential to provide a good-quality intermediate vision. While decentration decreased the optical quality of both models, tilt resulted in less significant changes that became more pronounced with increased aperture. The decentration amount tested in this study (0.50 mm) exceed what has been clinically reported for the Clareon (0.26 ± 0.13 mm)^17^ and Tecnis platform (0.27 ± 0.15 mm)^18^. Therefore, the potential impact observed may be considered a worst case for typical clinical decentration. Both lenses were similar in introducing (minimal) spread of the halo pattern.

While no literature on clinical studies is yet available for Clareon Vivity, there are many studies that have evaluated and compared the predecessor model, AcrySof IQ Vivity, with monofocal, trifocal, and other EDoF IOLs in clinical and laboratory studies. Our predicted VA of − 0.04 logMAR value at far distance agrees with Arrigo et al. in their prospective study of 54 patients implanted with AcrySof IQ Vivity found a corrected distance VA (CDVA) of 0.0 ± 0.03 logMAR^19^ and Kohnen et al. in their prospective study of 16 patients with AcrySof IQ Vivity implantation reported a CDVA of − 0.02 ± 0.068 logMAR^20^. It was also similar to the laboratory study by Azor et al. where the Vivity model was compared to other EDoF IOLs including LuxSmart (Bausch & Lomb, Rochester, NY, USA), Tecnis Eyhance (Johnson & Johnson Vision Inc., Irvine, CA, USA) and Isopure (PhysIOL sa/bv, Liege, Belgium)^21^. They found all lenses showed an expected VA of 0.0 logMAR at the far distance^21^.

In the intermediate range, our Clareon Vivity IOL results of 0.05 logMAR at 67 cm were better than those reported by Shafer et al. in a prospective multicenter trial that compared the long-term outcomes of the AcrySof IQ Vivity to a monofocal control. They found patients implanted with Vivity to have a better mean binocular uncorrected intermediate VA (UIVA) at 66 cm (0.18 vs. 0.29 logMAR) compared to the ones implanted with the AcrySof IQ Monofocal SN60WF, (Alcon, Fort Worth, TX, USA)^22^. However, these results were in disagreement with Kohnen et al. where distance corrected intermediate VA (DCIVA) measured at 66 cm was 0.10 ± 0.085 logMAR^20^. In another similar prospective randomized controlled study, Schallhorn et al. compared the visual outcomes of 107 patients implanted with the AcrySof IQ Vivity to 113 patients implanted with the control Acrysof monofocal IOL (Acrysof IQ SN60WF)^23^. They found that the EDoF IOL provided non-inferior monocular CDVA and better monocular DCIVA compared to the monofocal control. In the study by Arrigo et al., UIVA was reported to be 0.05 ± 0.03 logMAR at 80 cm, similar to our finding of 0.02 logMAR at 80 cm^19^. In our previous laboratory study using the same optical-set up as this study, we similarly found AcrySof IQ Vivity to expand the intermediate range by approximately 0.75D over the monofocal Tecnis ZCB00 (Johnson & Johnson Vision Inc., Irvine, CA, USA), with an expected gain of 0.2 logMAR^24^. Lastly, in respect to our estimated VA at near focus of 0.28 logMAR, our results agree with those of Kohnen et al. where distance corrected near VA (DCNVA) was reported 0.29 ± 0.106 logMAR at 40 cm^20^, and with the Schafer et al. study where the uncorrected near vision was found to be 0.30 logMAR in 15 patients implanted bilaterally. Overall, it appears that our findings of expected VA of Clareon Vivity IOL agree with most clinical and laboratory studies of AcrySof IQ Vivity IOL at near, intermediate, and far distances, showing almost 20/20 vision performance at the intermediate distance - underlining the validity of our approach.

Our laboratory predictions of optical quality do not always agree with the findings in clinical studies. In an early clinical study on the PureSee by Corbett et al., 60 subjects received the ZEN00V model bilaterally and 57 patients received the ICB00 as the control group^25^. Randomization was applied to both groups. In that study, monocular DCNVA (40 cm) was 0.37 ± 0.10 logMAR, showing a 0.06 logMAR improvement over the control, representing a substantial deviation from the predicted 0.24 logMAR. Similarly in another laboratory setting, Alarcon et al. found VA was equal or better than 0.20 logMAR at − 2.2D^26^. Schmid and Borkenstein studied the optical quality of TECNIS PureSee (ZEN00V) IOL and they found that for the 3 mm aperture, a near continuous range of vision was detected with a simulated VA better than 0.1 logMAR (Snellen 0.8) until a defocus of − 2.25D, and better than 0.2 logMAR (Snellen 0.63) even until defocus of − 2.75D.^27^ In regards to the intermediate range, Corbett and co-workers reported monocular DCIVA (66 cm) of 0.13 ± 0.08 logMAR, which again falls short of the predicted 0.07 logMAR with our optical testing^25^. Alarcon et al. similarly reported an intermediate VA of approximately 0.04 logMAR at − 1.5D and Schmid and Borkenstein reported a value of approximately 0.08 logMAR at − 1.5D^26,27^. The Corbett et al. study also showed that the EDoF effects can be improved by a refractive correction strategy, evidenced in a small subset of patients from the same study^25^. These findings indicate that future studies are needed to increase the reliability of laboratory findings regarding EDoF VA predictions for clinical outcomes. Although, it is noteworthy to mention that our predictive models were derived from binocular data^26^, but the Corbett group reported only monocular results in their clinical study.

Corbett et al. and Black et al. reported a CDVA of − 0.06 logMAR, with a standard deviation of ± 0.07 logMAR or ± 0.08 logMAR, respectively^25,28^. These two publications summarize the results of one clinical trial conducted on the PureSee. The distance vision results obtained in the clinical evaluation appear to conform to our findings, which predicted − 0.05 logMAR postoperatively. This finding also agrees with the objective evaluation of Alarcon et al., who conducted a similar assessment of the PureSee but used a different optical setup and computer-based simulations^26^. Clinical and laboratory investigations demonstrated PureSee’s high tolerance to low uncorrected refractive error and robust performance under natural levels of higher-order aberrations^26,28^. Although a broad defocus curve of the Clareon Vivity may prove advantageous in managing a good optical performance in conditions of ± 0.50D spherical or 0.75D cylindrical defocus, more research is needed to confirm this conjecture. SA correcting IOLs have generally been considered more susceptible to quality loss when placed in an off-axis position. Although PureSee features higher SA correction, the effects of IOL misalignment we observed were close between the two models.

Applying the refractive principle may also lower the incidence of photic phenomena. For the Vivity, 63% of patients reported no optical phenomena^20^. While there are no studies on Clareon Vivity, multiple prospective studies on patients with AcrySof IQ implantation reported no photic phenomenon, such as glare or halo or starburst, at a rate ranging from 80 to 100% at follow-ups^29–32^. In regard to PureSee, 60% of the patients did not report experiencing halos, and 72% and 76% did not perceive starburst and glare, respectively, by the Corbett et al. study^25^. Alarcon et al. revealed that the refractive TECNIS PureSee IOL showed a lower level of dysphotopsia compared to the diffractive TECNIS Symfony IOL, and similar levels to a monofocal IOL in a laboratory study^26^. Overall, our laboratory investigation confirms the comparable light spread between the two refractive models.

Evaluating higher-order aberrations under lens misalignment could provide a more comprehensive understanding of the effects of tilt and decentration on IOL performance. Therefore, the absence of this analysis, due to the unavailability of a wavefront metrology device, can be considered one of the limitations of our study. Future research on the type and magnitude of aberrations introduced by the two EDoF models in relation to various degrees of misalignment and pupil sizes is needed to help elucidate the complex relationship between IOL misalignment and its effect on the optical quality. Another limitation of our study in predicting clinical outcomes is the strictly controlled conditions in the laboratory testing. The testing also fails to reflect the variation in outcomes that exists between patients due to ocular biometry differences between individuals. Despite these limitations, our study results can serve as a benchmark until more clinical data becomes available.

In conclusion, this laboratory investigation demonstrated that the Clareon Vivity and the Tecnis PureSee can improve patients’ visual function at intermediate distances. Overall, the performance of the tested IOL models, including tolerance to misalignment and potential to induce photic phenomena, showed similar predicted effectiveness.

## Methods

### Intraocular lenses

The Clareon Vivity (model CNWET0 and CNAET0 for the pre-loaded version) is made of Clareon hydrophobic acrylic material with a 1.55 refractive index and an Abbe number of 36.3 ± 0.7 (Supplementary Table S1)^33^. The aspheric design of the Vivity corrects − 0.20 μm of primary SA^34^. It is planar with an optic diameter of 6.0 mm and overall length of 13.0 mm^34^. Its power is available in a range from + 10.0 to + 30.0D in 0.5D increments^34^. The extension of the visual range to the intermediate region is achieved through non-diffractive Wavefront-Shaping Technology (X-Wave technology). The Vivity features the central 2.2-mm area with two transition zones having different aspheric-surface profiles. The inner transition element directs the light towards the near end of the defocus extension. The base-lens surface, which separates the first and second surface transitions, contributes to the distance image. Light passing through the second transition element reinforces the mid-intermediate range to create an EDoF effect.

The Tecnis ZEN00V PureSee and its pre-loaded version (DEN00V) are recent introductions to the refractive EDoF category. Similar to other Tecnis models, the material is hydrophobic acrylic that has a refractive index of 1.47 and an Abbe number of 55. It features a biconvex design, with a wavefront-modified anterior aspheric surface to compensate for the average level of corneal SA^35^. This IOL has a 6.0 mm optic diameter and is 13.0 mm in overall diameter^36^. Its available diopter power ranges from + 5.0 to + 34.0D in 0.5D increments^36^. The PureSee introduces the depth-of-focus extension by applying a power profile change^26^.

Two samples from each model were evaluated, all with a refractive power of + 20D.

### Optical setup

The optical quality of the IOLs was measured using the Laboratory’s OptiSpheric IOL PRO2 (Trioptics GmbH, Wedel, Germany) per the ISO 11979-2 standards, using a light source, a reticle, a collimator, an eye model, a microscope, and a charge-coupled device camera^37^. Given this method, the effective focal length was measured with a tolerance of ± 0.3%, and the MTF was measured within ± 2%.

### Image quality metrics

The quality of image projection by the IOLs was evaluated via the MTF calculated by the Fourier transform of the line spread function, using polychromatic light and a model cornea (+ 0.27 μm of SA at 5.15 mm), at both 3- and 4.5-mm apertures^37,38^. A 50 lp/mm frequency was used as a quality criterion to determine the MTF. The average MTF value, corresponding to the MTFa metric was calculated at a range of 1 to 50 lp/mm.^39^ MTFa is highly correlated to VA in patients with monofocal and presbyopia-correcting IOLs. In this study, VA was calculated as outlined below and according to ANSI Z80.35-2018 (a = 0.085, b= − 1.0, and c= − 0.21).

$$VA = a \cdot MTFa^{b} + c$$

The MTF was graphed up to 100 lp/mm, with TF MTF measured at 25, 50, and 100 lp/mm with a defocus range of + 1.0 to − 2.5D at the spectacle plane (0.125D step). To subjectively assess the optical quality of the lenses, the 1951 USAF resolution test chart was recorded at 3 mm.

### IOL misalignment

The IOL’s tolerance to misalignment – decentration or tilt – was tested by assessing the TF MTF. Decentration was induced at 0.50 mm, and a 5° tilt was simulated using a dedicated insert provided by Trioptics GmbH.

### Characterizing unwanted visual effects

To identify the spurious energy of the lenses, we used a polychromatic PSF to compare their light distribution at the 4.5 mm aperture beyond the PSF center^40,41^. Although the OptiSpheric IOL PRO2 has an 8-bit camera, its dynamic range was extended by combining images of different shutter times. The PSF cross-sections were plotted on a logarithmic scale, as the function of the normalized light intensity over a visual angle in arcmin.

### Statistical analysis

Measurements were performed with three repetitions and averaged. Data was analyzed using MATLAB (Mathworks, Natick, MA, USA).

## Electronic supplementary material

Below is the link to the electronic supplementary material.

Supplementary Material 1

## Data Availability

The datasets generated during and/or analyzed during the current study are available from the corresponding author on reasonable request.
