# Supplementary material for: Evaluation of Clareon Vivity and PureSee intraocular lenses: optical quality, depth of focus and misalignment effects
Source: Sci Rep. 2025 Jul 24;15:26943. doi: 10.1038/s41598-025-07970-y (PMC12290095; doi:10.1038/s41598-025-07970-y)
Supplement: Supplementary file 1 — Supplementary Material 1 [file 41598_2025_7970_MOESM1_ESM.docx]

**Evaluation of Clareon Vivity and PureSee EDoF IOLs: optical quality and its association with misalignment and spectral and spherical-aberration conditions**

Ava Niknahad, Zhiyi Wu, Hyeck-Soo Son, Gerd U Auffarth, Ramin Khoramnia, Grzegorz Łabuz

| **Intraocular lenses** | **Manufacturer** | **Powers** | **Optic and Overall Diameter** | **Material** | **Index of Refraction** | **Abbe number** | **Sphericity** |
| --- | --- | --- | --- | --- | --- | --- | --- |
| Clareon Vivity (CNWET0) | Alcon Laboratories | +10.0D to +30.0D in 0.5D increments | 6.0mm; 13.0mm | Hydrophobic acrylate/methacrylate copolymer | 1.55 at 35°C | 36.3 | -0.20 µm |
| Tecnis PureSee (ZEN00V) | Johnson & Johnson Vision Inc. | +5.0D to +34.0D in 0.5D increments | 6.0mm; 13.0mm | Hydrophobic acrylic | 1.47 at 35°C | 55 | -0.27 µm |

**Supplemental Table S1.** Summary of Clareon Vivity and Tecnis PureSee properties.
